# Faith-based leaders’ perceptions on the implementation of programs to promote healthy lifestyles in churches in Barbados-a mixed-methods analysis

**DOI:** 10.21203/rs.3.rs-4014464/v1

**Published:** 2024-03-06

**Authors:** Natasha Sobers, Madhuvanti Murphy, Saria Hassan, Katrina Norville, Lisa Brathwaite-Graham, Ian Hambleton, Simon G Anderson, Kia Lewis, Trevor Ferguson

**Affiliations:** George Alleyne Chronic Disease Centre, Caribbean Institute for Health Research, The University of the West Indies, Cave Hill Campus, Barbados; George Alleyne Chronic Disease Centre, Caribbean Institute for Health Research, The University of the West Indies, Cave Hill Campus, Barbados; Department of Medicine, Emory University School of Medicine, Atlanta, Georgia, United States; George Alleyne Chronic Disease Centre, Caribbean Institute for Health Research, The University of the West Indies, Cave Hill Campus, Barbados; New Testament Church of God, St. Michael, Barbados; George Alleyne Chronic Disease Centre, Caribbean Institute for Health Research, The University of the West Indies, Cave Hill Campus, Barbados; George Alleyne Chronic Disease Centre, Caribbean Institute for Health Research, The University of the West Indies, Cave Hill Campus, Barbados; George Alleyne Chronic Disease Centre, Caribbean Institute for Health Research, The University of the West Indies, Cave Hill Campus, Barbados; Epidemiology Research Unit, Caribbean Institute for Health Research, The University of the West Indies, Mona Campus, Jamaica

**Keywords:** acceptability, appropriateness, Small Island developing states, faith-based organizations, chronic disease self-management

## Abstract

**Background:**

There is a high burden of chronic diseases such as hypertension and diabetes in small island developing states (SIDS). SIDS governments have committed to a range of public health, healthcare, and fiscal measures to reduce this burden including community-based health education in collaboration with civil society organizations. We sought to explore perceived acceptability, appropriateness, and feasibility of implementing self-management health programs in 20 faith-based organizations in the small island developing state of Barbados.

**Methods:**

This was a concurrent mixed methods study - a quantitative online survey and a qualitative inquiry using semi-structured interviews. Acceptability, appropriateness and feasibility of the intervention were assessed using the following quantitative assessment tools: Acceptability of Intervention Measure (AIM), Intervention Appropriateness Measure (IAM) and Feasibility of Intervention Measure (FIM). Thirteen in-depth interviews were conducted virtually, recorded and transcribed verbatim. Transcripts were analyzed using thematic analysis based on deductive codes from Proctor’s implementation outcomes definitions.

**Results:**

From the 52 respondents of the survey, the median and interquartile ranges for the AIM, IAM and FIM scales were 16 (15–20), 16 (16–20) and 16 (15–17) (out of 20), respectively. We found high levels of acceptability, 82% (95% CI (69%, 95%)) of leaders indicating that health programs in churches met with their approval; and high levels of appropriateness-90% (95% CI (80%, 100%)) indicating health programs in churches were “fitting” and “a good match”. Feasibility scores were lower, with 60% (95% CI (44%, 76%)) indicating that health programs in churches would be easy to use. In interviews, leaders expressed acceptance of healthy lifestyle programs in churches and described their appropriateness through alignment with church doctrines stating, “the body is the temple of God”. They felt that economic impacts from COVID-19 were likely to be a barrier to the success of programs. Leaders expressed the need for support from healthcare providers who are sensitive and respectful of church culture.

**Conclusion:**

We found that health-based programs in churches align well with church doctrines, but the success of these programs will depend on establishing trust through the engagement of church-based champions, tailoring programming to include a biblical perspective and engaging entire households.

## Introduction

There is a high burden of chronic lifestyle-related diseases such as hypertension and diabetes in small island developing states (SIDS)(1–3). SIDS governments have pursued fiscal policies, public health and healthcare realignment programs and community involvement to decrease the Non-communicable Disease (NCD) burden including the involvement of civil society organizations (CSOs)(4, 5). Government partnerships with CSOs like service organizations (e.g. Kiwanis and Rotary Club), patient advocacy groups and faith-based organizations have included both health education as well as programmatic intervention. Faith-based organizations (FBOs) have played an important role in the delivery of cardiovascular risk factor reduction programs that have been particularly useful for populations with health disparities(6). In a recent systematic review, church-based programs were effective in two-thirds of weight reduction and blood pressure control studies and half of physical activity and diet risk factors(6).

In the Caribbean, religion continues to be an important cultural reality with approximately 90% identifying as belonging to a religion(7). Some work has been done to explore feasibility of health programming (8) in churches in the Caribbean but the literature remains limited particularly in understanding the views of church leadership on the implementation and delivery of these programs.

Caribbean governments have expressed commitment to adopting and adapting the Chronic Care Model (CCM)(9) to enhance the management of NCDs and many have also adapted and adopted the HEARTS in the Americas initiative (10). One of the major components of the CCM and HEARTS is community-based programming to promote self-management educational initiatives which are often delivered through partnerships with CSOs(11). These health programs emphasize self-management education as key components of care, and several Caribbean SIDS have begun to implement self-management programs through partnerships with faith-based organizations and other CSOs(12, 13). Several countries have attempted to implement self-management programs, but we found few reports or peer-reviewed publications to date on any aspect of the implementation of these programs.

Given the impetus by local, regional, and international health entities to partner with CSOs to implement self-management and other health education programs, we explored perceived acceptability, appropriateness, and feasibility of implementing self-management health programs in churches in Barbados. Prior studies have linked FBO-based programming in Barbados with implementation effectiveness (14). We also explored perceived barriers and facilitators to implementation from the church leaders’ perspectives with the aim of informing an implementation strategy. In this study we use the terms church and faith-based organizations interchangeably and because of the pervasiveness of religion in Caribbean culture we use the church as an example of a civil society organization with which health entities may wish to partner. This mixed methods exploration preceded the implementation of a study to examine the impact of Chronic Disease Self-Management Program on blood pressure values (15).

## Methods

### Study Design and Setting

This was a concurrent mixed methods study consisting of a quantitative online survey followed by a qualitative inquiry using semi-structured interviews. We conducted the study in 2021–2022 in 20 churches in Barbados, a country where 80% of the population identify as belonging to a religion(16). Barbados is classified by the World Bank as a high-income small island developing state where the population of approximately 287,000 consists of 92% Black, 3% White, 3% Mixed and 1.5% Indian descent (16). Historically, religion has played an important role in the development and socio-political landscape of the island. Religious clergies are represented in the Senate section of Parliament, on Boards of Management of schools and other major sectors as well as playing active role in the National Non-Communicable Disease Commission. Christianity is the predominant religion, and the island is home to at least 15 Christian denominations.

### Quantitative phase: Data collection

In this paper, the term ‘church leader’ refers to a person who leads and manages a single assembly (church) usually ranging in membership from 30 to 200 persons. All these persons held the titles of either reverend, pastor, or priest. Before the questionnaires and interviews, church leaders were given a 30-minute presentation on the main concepts and activities found in the chronic disease self-management program (CDSMP) developed by Stanford and administered by the Self-Management Resource Centre (17). CDSMP is delivered in six-week workshops aimed at enabling participants to manage their chronic disease(s) effectively (18). The presentation to church leaders included a description of the planned implementation project which would include churches in the implementation of this program. This plan has been described more fully in a previous publication describing the protocol for a trial to be conducted in FBOs in Barbados (15). Having received the description of the program, a REDCap link was sent for completion of the quantitative survey and church leaders were selected for semi-structured interviews. The link was shared on the Zoom platform and via WhatsApp to church leaders.

### Quantitative phase: Data tools and analysis

Using the Acceptability of Intervention Measure (AIM), Intervention Appropriateness Measure (IAM) and Feasibility of Intervention Measure (FIM)(19), we assessed acceptability, appropriateness, and feasibility of implementing healthy lifestyle programs in churches. The AIM, IAM, and FIM scales each have four items and in other settings have been tested for and shown to have good content validity, discriminant content validity, reliability, structural validity, structural invariance, known-groups validity, and responsiveness to change (19). These constructs used together have been found to be particularly relevant to the adoption of an intervention (20). Additionally, we used a modified version of the Organizational readiness for implementing change-adapted to the context of the church(21). For each item on the scales, participants were given the option to respond “1-Completely disagree, 2-Disagree 3-Neither agree nor disagree, 4-Agree 5-Completely agree. We calculated and reported the proportion of persons who responded completely agree/agree with 95% confidence intervals. We calculated mean scores and 95% confidence intervals for each item on the questionnaire and internal consistency (using Cronbach’s alpha) for each measure given that this is their first usage in this population. Analyses were conducted using Stata Statistical Software (22).

### Qualitative phase: Data collection and analysis

For the qualitative interviews, church leaders were purposively selected from four denominations because these were the denominations who replied to our request for corporate presentations with pastors. Thirteen in-depth interviews were conducted virtually via Zoom with church leaders because of the COVID-19 pandemic. They were recorded with consent and transcribed verbatim. Our interview guide is attached and demonstrates that our questions to pastors included but were not specific to chronic disease self-management programs. We asked questions widely about education and training on healthy lifestyle programs in churches.

Transcripts for church leaders’ semi-structured interviews were analyzed using thematic analysis informed by both inductive and deductive coding. The latter were derived from Proctor’s definitions of the implementation outcomes-acceptability, appropriateness, and feasibility. Using Proctor’s definitions as the basis for our understanding of the three implementation outcomes, we defined our three main constructs as described in Table 1 which also outlines our coding framework and the way in which the terms were operationalized for this study (23). In reviewing church leaders’ interviews we considered their perception of their own acceptance as well as their perception of the acceptance of church members.

All documents were coded by the first author NS. To ensure validity, codes were reviewed by senior qualitative researcher MM for the first three transcripts and coding framework updated to include the inductively obtained codes and to better explain the definitions being used for feasibility, acceptability and appropriateness. Fifty percent of transcripts were double coded by co-authors KN and LBG and analyzed by the three coders and senior qualitative researcher. The coding team are all public health researchers and members of faith-based organizations who reside in Barbados. We applied deductive and inductive thematic analysis to the transcripts until data saturation had been reached. We used a convergent parallel design in analyzing results from the two arms of our study. The quantitative and qualitative results were analyzed separately, and we then compared the two arms and summarized areas of convergence and divergence.

This study was given approval by the University of the West Indies/Ministry of Health and Wellness ethical review board of Barbados (Reference no: 2000504-B-IRB).

## Results

### Description of participants

Of the 107 church leaders to whom the survey was sent, 52 consented and attempted the survey and 40 (39%) responses contained sufficient data for analysis. The median and interquartile ranges for the AIM, IAM and FIM scales were 16 (15–20), 16 (16–20) and 16 (15–17) (out of 20), respectively. On examining individual items, highest percentage of pastors agreed with the item-“Chronic Disease Self-management program in church seems possible” (97%) and the lowest percentage was found for the item where leaders responded to the statement: “Chronic Disease Self-management program in church seems easy to use” (60%). Given that this was the first use of the AIM, IAM and FIM questionnaires in this population, we assessed the internal consistency of the scales using Cronbach’s alpha and the scores were as follows: 80% - AIM; 96% - IAM; 82% - FIM.

For the semi-structured interviews, we conducted thirteen in-depth interviews with church leaders from five denominations-New Testament Church of God, Wesleyan, Anglican, Nazarene, and Independent Pentecostal. There were nine males and four females interviewed ranging in age groups from 35 to 75 years old.

### Acceptability

The quantitative survey showed high levels of acceptability, between 77 and 92% of respondents indicating that the self-management program as described to them would be acceptable within the church (Table 2). This assessment was in alignment with the overall findings from the semi-structured interviews. Church leaders generally indicated that it would be acceptable to them but held mixed views around how acceptable the program might be to their members.

### Acceptability: church leaders describe palatability of health programs

Mirroring the quantitative findings, church leaders were very positive about acceptability of self-management programs within the church. Church leaders consistently referred to the role the church needed to play in promoting healthy lifestyles.

Some leaders highlighted their acceptance of the integration of healthy lifestyle programs within the church by providing examples of those that already existed:

*“We have many presentations for health. We look at different areas of the Women’s Health, men’s health and the total man. We had an exercise program but because of COVID that has stopped a little bit.*” (EA, Female, 50–60 yrs)

*“We had a chef who is one of our members and he started teaching about healthy living and healthy cooking… how to do your chicken and so on without frying and trying to do your vegetables and you know steaming and so on.”* (TS, Female 60–70 yrs)

Many leaders also saw the church as having a health promotion role through making education/advice and diagnostic checks more readily accessible to members:

*“Some persons have an aversion to going to a doctor um…or don’t have a lot of patience going to the clinic, um… Whatever the reason might be so here’s an opportunity to come and get some of those things checked without having to pay any money without any hassle whatever the care maybe and then you get back the results and then you can you know be given guidance as to what to do from there.”* (IR, Male, 40–50 yrs)

### Acceptability: Pastors perception of members’ acceptability of health programs

While church leaders were confident in their own acceptance of health programs, they held mixed views on the likely acceptability to members. Only 63% (95% CI: 48%, 79%) felt that members were determined to implement healthy lifestyle changes in their own lives and 63% felt that their members feel confident that they can keep track of progress made in creating a supportive healthy environment. During interviews, leaders revealed that in their attempts to change the content of their sermons to include more health-related content, they may experience some “backlash”.

*“So like half, not half, like about a third of the service, every Sunday is going to be about health… So I know, um how some persons may say to me that they prefer to have the entire service on about God and Jesus…And less doing with seems spiritual, so I know I may get some backlash there in terms of that, but I know it may be a challenge.”* (WL, Male, 35–40 yrs)

While church members welcomed health education and screening activities there was resistance to programs that tried to introduce individual level care as some saw this as a replacement of their personal health care provider.

*“Because in their mind this is a medical situation. I already trust this particular doctor. I don’t need to back talk to the pastor about this. I will go to the doctor. We find that mainly that is what would happen.”* (IR, Male, 40–50 yrs)

Finally, as it relates to perceived members’ acceptance, church leaders felt that even those members who might be amenable to such a program might have difficulty in maintaining healthy lifestyle recommendations. These barriers identified within acceptance may affect the feasibility of implementation.

### Appropriateness: Healthy lifestyle programs are aligned with doctrine

The levels of appropriateness were high in both quantitative and qualitative assessments. Approximately 90% of church leaders believed these programs are appropriate (Table 3). In the interviews, they explained that health was a priority for the church as it deals with the whole man. Interestingly, some were keen to highlight that the church’s priority is spiritual health and thus that must take precedence over all else.

Church leaders consistently aligned healthy lifestyle programs with the tenets/doctrines of their faith irrespective of denomination. Leaders frequently quoted from various passages of scripture. Most commonly, pastors mentioned the concept that the body is the “temple” of God which should be treated with care. They also quoted from their own doctrinal commitments for example the Marks of Mission which express the Anglican community’s common commitment to, and understanding of, God’s holistic and integral mission.

*“Well the churches role should be to promote the healthy eating and physical activity um from a biblical standpoint, if I may use that because the body is the temple and we have been commissioned to take care of the temple.”* (OE, Male, 30–40 yrs)

*“The body was created by God. It’s good. and Part of our responsibility as the Psalmist tells us he made us a little lower than the angels to take care of god’s created order and part of what was created is the body. from managing illnesses, managing ourselves is part of that role.”* (GF, Male, 40–50 yrs)

### Appropriateness: Health programs appropriate but spiritual health is the priority

Similar to what was described when addressing the acceptability of chronic disease self-management programs in churches, the issue of spirituality being seen as the priority of the church was discussed:

*“The primary health concern is the spiritual health, of course we must also be concerned with our physical health, our mental health, or emotional health The major challenge that I see… when we would do it. Do we sacrifice like our bible study time or pray time*, (IR, Male 40–50 yrs)

### Feasibility: pandemic effects on health programs in churches

For three of the four quantitative items, more than 85% of church leaders indicated that chronic disease self-management programs in churches seem implementable, possible and doable (Table 4). Only 60% thought that such a program would be easy to use/follow. In semi-structured interviews, church leaders shared their ideas on the feasibility of the programs in churches by drawing on experiences they had had previously with health programs in churches. Based on these experiences, they were able to share potential barriers and provide advice on possible facilitators. They spoke about the pursuit of health and wellness activities such as hiking and health fairs; however, the Covid-19 pandemic hindered many of these health-related activities” creating a barrier to positive health practices such as physical activity and social interaction.

Some church leaders felt that the economic impact of the pandemic, specifically the loss of income due to COVID-19 contributed to poor dietary habits in people, because of the lack of affordability of nutritious foods considered to be more expensive.

*“Due to COVID etc, a lot of persons are no longer working. Finances have become a problem, and healthy eating is expensive unfortunately.”* (OE, Male, 30–40 yrs)

This COVID-19 impact is likely to continue beyond the pandemic for both the individual and the church as an organization.

### Feasibility: the relationships between food, culture, and church fundraising events

As is culturally typical in Barbados and the wider Caribbean, big events such as church fundraisers are food-centered events. Many foods high in salt, fat and sugar are also culturally accepted foods that are expected to be sold at these events to attract patrons. Anecdotally, the relationship between these foods (e.g. coconut sweetened bread, macaroni pie-a cheese filled pasta dish, and fish cakes) and fundraising is strong; healthy items have less selling power. Yet having such items at church events would undermine chronic disease self-management programs.

*“At church events we sell a variety of things ……we do healthy foods we have those people who would promote healthy foods, but you know the Barbadians still like them fishcake (fried fish and flour)… and them souse (pickled pork) and those kind of things so we cater to them. We have fruit, we have vegetables we have fruit drinks, we prefer not to use the… bottle drinks and you know the drinks like the sodas.”* (AL, Female, 70–80 yrs)

There is a fear that removing unhealthy items for sale would challenge the financial success of such events and the church financial bottom line.

### Feasibility: church leaders not equipped to deliver health-related topics

Despite acknowledging the appropriateness of health programming within the church, some leaders expressed that they felt ill equipped to address health-related topics themselves.

*“I know how to preach the word but I don’t know how to ask people about how they are feeling regards with regards to disease and diabetes and hypertension so putting someone in place like that to be able to communicate with them is important”*, (OL, Male, 40–50 yrs)

Many indicated the need for a focal point/health champion within the church with the knowledge on how to communicate these issues appropriately, to assist in this regard.

### Feasibility: facilitators to health programs in churches

Church leaders shared ways that programs can be shared to make health programs in churches more effective:

1. Use a Biblical perspective: Our respondents felt that using biblical verses and examples would enhance the adoption of the program within churches and increase its success.
  *“And I find when you are dealing with um Christians, you have to bring a biblical perspective. Why they should not do this and shouldn’t do that and, you understand?......... Especially when you are dealing with the older folks”* (AR, Female, 60–70 yrs)
2. Build and maintain trust: There was a sense that persons were generally skeptical of some health information and of taking medications. Leaders therefore suggest that before health information is shared, collected and used, researchers and health educators should work on building the trust of congregants.
  *“So I think um… the challenges is to gain people’s confidences that you are not going to be um… the information you get will be used confidentially.”* (AE, Male, 60–70 yrs)
3. Keep materials and questionnaires simple and avoid jargon: This was an important theme for the church leaders who felt that some of the information presented was not always at a level that members and the community could understand. Thought must therefore be given to the health literacy of the society being served.
  *”Where possible you have a simple questionnaire and probably make it easier …for people to respond. That’s why I said simplicity in the questionnaire. So that’s another thing, the language that … is used … I know you can’t really adulterate the terms but if it can be simplified it would help.”* (AE, Male, 60–70 yrs)
  *“So in other words being able to use less jargon but not water down the facts of what you are sharing.”* (IR, Male, 40–50 yrs)
4. Individual and committee champions needed: As seen in other organizations, pastors here felt that health champions should be identified to lead if the effort is to be successful, but some felt that they may experience challenges in finding persons interested in taking up this role.
  *“We would have to put some person in charge of it, yes, we have church clerk and we have you know the normal church structure, ……you would have to put somebody in charge of it to be responsible for it and make sure that everything is recorded and given to the Church. Cause to go and put this on um on the system now, it will be too much.”* (GF, Male 40–50 yrs)
  *“Finding the person might be the hardest part now to want to take up that role.”* (RE, Male, 40–50 yrs)
5. Programs should offer practical solutions
  They encouraged doctors and other healthcare providers to provide practical solutions to the health problems they identify since simply outlining the problem with no solution can be “depressing”.
  *“Just you know practical ways in which the particular cause I know sometimes from sessions not at our church but I’ve been to sessions before that people literally left depressed cause it was like okay so I am dead. There is nothing. I just heard about a million things that are probably wrong with me and …there was no solution given right?”* (IR, Male, 40–50 yrs)
6. Create family programs: There was the recognition by some of our leaders that these conditions can occur and may be caused by behaviors that occur at various stages of life. Thus, they called for an approach to healthy lifestyle programs that considers integration of the whole family.
  *“Put a spin on it so that it is family oriented,……. So get the whole family involved. Because these matters...these issues. I do believe there, there are some children some teenagers, who are afflicted with these illnesses as well.”* (GF, Male, 40–50 yrs)
  *“We’ve done a lot on families as well. Because strong families for me builds strong communities and strong churches”* (TS, Female, 60–70 yrs)

### Feasibility: Churches need health professional support to deliver programs

Even though church leaders believe that churches can create a facilitative environment for behavior change through supporting of health programs within the church delivering health messages from the pulpit, some did not feel equipped enough to deliver the messages themselves. They described that they have invited health professionals to deliver these messages and indicated ways in which the professionals can improve their delivery and the appeal of the health message. While they have used professionals of different faiths/beliefs their preference was for persons of the same faith who understood the culture of the faith. One pastor indicated that he prefers to use the doctors from his own congregation because they “know the people” and the congregation “has confidence in them”-indicating the value of relationship, trust and the power of knowing the culture of specific faith.

### Reaching the wider community through churches

In the quantitative survey we asked pastors about their perceptions of the church’s readiness to be a community hub for healthy lifestyle programs. The majority of church leaders (90%, 95% CI 81, 99) felt that people who attend their church wanted to promote healthy living and 83% (95% CI, 72%, 95%) felt confident that the church could serve as a community hub for healthy lifestyle programs; From our inductive coding, a strong theme emerged around the impetus of the church organization to reach out to their neighboring communities for special projects and events. Church leaders freely discussed their activities in the communities explaining the extent to which the church could be used not simply as a mode to improve members’ health but as a vehicle by which the wider community around the church can be reached. Leaders viewed community involvement and outreach as part of their mission as churches and described it as aspects of their “compassionate” ministry.

*“Well sometimes when we have activities we don’t only restrict it church members. We would have suppose… there is a seminar we would encourage persons to invite their family and friends um… who are obviously not members of the church as well, so in a way you are helping them in reaching out in evangelism but at the same time we also reacting to a need that they would have so that…cause I don’t think as I said that the church is there just to preach at people…”* (AE, Male, 60–70 yrs)

The communities are more likely to attend outdoor church events like community fairs which involve but are not exclusive to health activities.

*“As part of our evangelistic effort um… every summer we had a major outreach in the (Community name) and we would setup tents and up jumping tents you know all these things and we would have a tent where you would get the same um… health checks being done, blood pressure checks, checking of heart rate, these different things happening there. So in that instance that would be an annual exercise.”* (IR, Male, 40–50 yrs)

In addition to community fairs, leaders mentioned other health-related community activities that included creation of kitchen gardens with children, exercise/gym activities on the church premises and line dancing.

Churches have engaged the communities via a variety of mechanisms from individual level family to family invitations, knocking on doors of houses within the communities to more public communication using flyers placed in mini-marts and local gas stations and social media platforms like WhatsApp, Facebook, and Instagram. The use of virtual platforms has increased access to those who did not previously attend. On the virtual platforms, church leaders have noticed more community/non-member attendees.

## Discussion

We found that health-based programs in churches have a high chance of being successful because of doctrinal alignment with church tenets but that the success of these programs will depend on establishing trust through engagement of church-based champions, tailoring programming to include a biblical perspective, and engaging the entire family. Success of programs will also require strengthening health-related capacity of FBO and providing necessary support to deliver health-related programming,

Our work expands the use of implementation tools (AIM, IAM and FIM) in assessing likelihood of health program adoption to a Caribbean FBO community in the pre-implementation phase and demonstrates high reliability of these instruments. Both our quantitative and qualitative findings suggest that health programs are perceived as appropriately aligned with the doctrines and mandate of the church. Church leaders expressed their acceptance of healthy lifestyle programs in churches but held mixed views on the perceived acceptance of their congregants/members. This perceived alignment of health and church programs has been mirrored in church studies in African American and Latino churches across the United States (24–26).

Church leaders are believed to have significant influence over their members and are thus poised to influence health behavior (17, 27–29). Therefore, some authors have recommended that one of the key elements for community-based health programs identified is commitment from church leaders to provide health education to the congregation (30, 31). In our study, the church leaders felt that they would need considerable support from health care providers to adequately deliver sustainable health promoting programs in churches. They specifically expressed a preference for health care providers who understand and are respectful of their church culture. This perhaps signals the need for closer collaboration between church and health system in this setting.

### Implications for Health Program Implementation in FBOs

Church fundraising is a potential source of unhealthy food availability for congregants and the community. Attempts to introduce health programs would need to be sensitive to this challenge. Alternatively, fundraising could be centred around activities other than food to reduce the temptation of having high salt, high fat, high sugar, items as currently occur. In our study, churches reported having health walks and it may be advisable for them to also include hikes, line dancing and other modalities that emphasize health. These church leaders reported significant activity within the community which can be leveraged to promote health programs. This could result in strengthening also for the church itself as it can be incorporated into evangelistic outreach.

This study was conducted during the COVID-19 pandemic and thus the pandemic public health measures had a significant impact on the church leaders’ perception of barriers to delivery. The economic factors which leaders felt might be barriers to healthy eating even prior to pandemic were exacerbated during this time. Other studies highlighted the financial challenges members experienced in attempting to live healthier (32). During the pandemic, the World Bank reported that “the crisis had a dramatic impact on global poverty and inequality”(33). Studies in Thailand and Philippines found that COVID-19 pandemic impacted the affordability of healthy diets particularly for the poor through a reduction in their incomes(34). These global perspectives are reflected in the observations of church leaders in this study.

### Strengths and Limitations

Our study used robust measures and constructs of acceptability which has not always been the case in the literature and is rare in Caribbean implementation studies. Acceptability, appropriateness and feasibility have together been used to predict adoption and our use of proctor’s implementation outcomes in a mixed methods study is not common in low-middle income or small island developing countries. Faith-based organization membership has a high proportion of women and thus our learnings from this setting may reflect that inherent organizational bias. We did, however, interview more men than women since there are more male leaders in the denominations sampled than female leaders.

To expand the reach of healthy lifestyle programs and promote equitable delivery, partnerships must be wider and include sports, patient led and other community-based organizations. We had a low response rate in the quantitative survey and those who responded may have been more likely to have an interest in healthy programs and thus gave what they perceived to be favorable responses. Some of the information from this study may be transferable to other CSO settings but further work in one or more of these settings is needed to understand the differences and to correct the gender bias in recruitment. This study was limited to the church leaders’ perceptions and did not examine the views of members on appropriateness, acceptability, and feasibility of health programs in the church organization.

## Conclusion

Given the World Health Organization’s endorsement of the integration of FBO communities in achieving the Sustainable Development Goals, studies such as this one can assist SIDS in understanding transferable lessons learnt in health program implementation. High acceptability and appropriateness indicate that health programs in churches in Barbados are likely to be successful. However, program planners and policy makers should take note of the need to establish trust and tailor programs to include a biblical perspective and allow these and other factors highlighted by church leaders to inform and guide future activities.

## Figures and Tables

**Table 1:** Theory-based coding framework

Coding name	Abbreviation	Theoretical Definition	Operational Questions
Acceptability	Acc	The perception that the health programs are agreeable, palatable or satisfactory for the church	Will people accept these programs in the church setting?
Appropriateness	App	Perceived fit, relevance or compatibility of the health program.	How well does the health program fit with the tenets/doctrines of the church?
Feasibility-facilitators	Fea-f	The extent to which health programs can be carried out in the church setting	What are the facilitators to implementing health programs in FBOs?
Feasibility-barriers	Fea-b	The extent to which health programs can be carried out in the church setting	What are the barriers to implementing health programs in FBOs?

**Table 2:** Mean and proportional scores for each item on the Acceptability of Intervention Measure scale

Survey items	Mean (95% CI)	Percentage who agree/strongly agree%, (95% CI)
**Acceptability of Intervention Measure**		
Chronic Disease Self-management program in church meets my approval.	4.0 (3.7,4.3)	82 (69, 95)
Chronic Disease Self-management program in church is appealing to me.	3.9 (3.6, 4.3)	77 (63, 91)
I like Chronic Disease Self-management program in church.	4.0 (3.7, 4.3)	82 (69, 95)
I welcome Chronic Disease Self-management program in church.	4.3 (4.0, 4.5)	92 (83, 100)

**Table 3:** Mean and proportional scores for each item on the Intervention Appropriateness Measure

Survey items	Mean (95% CI)	Percentage who agree/strongly agree%, (95% CI)
Chronic Disease Self-management program in church seems fitting.	4.3 (4.0, 4.6)	90 (80, 100)
Chronic Disease Self-management program in church seems suitable.	4.2 (3.9, 4.5)	88 (77, 98)
Chronic Disease Self-management program in church seems applicable.	4.3 (4.0, 4.6)	90 (80, 100)
Chronic Disease Self-management program in church seems like a good match.	4.3 (4.0, 4.5)	90 (80, 100)

**Table 4:** Mean and proportional scores for each item on the Feasibility Intervention Measure

Survey items	Mean (95% CI)	Percentage who agree/strongly agree%, (95% CI)
Chronic Disease Self-management program in church seems implementable.	4.1 (3.9, 4.3)	88 (77, 98)
Chronic Disease Self-management program in church seems possible.	4.3 (4.1,4.5)	97 (92, 100)
Chronic Disease Self-management program in church seems doable.	4.2 (4.0, 4.4)	92 (84, 100)
Chronic Disease Self-management program in church seems easy to use.	3.6 (3.4, 3.9)	60 (44, 76)

## Data Availability

The datasets generated and analysed during the current study are not publicly available due to the increased probability of information being identifiable given the small size of the island population. The data are available from the corresponding author on reasonable request.

## Abbreviations

AIM: Acceptability of Intervention Measure
CCM: Chronic Care Model
CDSMP: Chronic Disease Self-Management Program
CSOs: Civil Society Organizations
FIM: Feasibility of Intervention Measure
FBO: Faith-based organization
IAM: Intervention Appropriateness Measure
NCD: Non-communicable Diseases
SIDS: Small Island Developing States
